# Placenta Accreta Spectrum with Ureteral Invasion due to Progression of Cesarean Scar Pregnancy

**DOI:** 10.1155/2023/9065978

**Published:** 2023-10-07

**Authors:** Nana Yara, Yoshino Kinjyo, Yukiko Chinen, Tadatsugu Kinjo, Keiko Mekaru

**Affiliations:** Department of Obstetrics and Gynecology, Graduate School of Medicine, University of the Ryukyus, Japan

## Abstract

Expectant management is not recommended for cesarean scar pregnancies because they are often associated with placenta accreta, cesarean hysterectomy, and massive life-threatening hemorrhages during delivery. Herein, we report a case of placenta accreta spectrum with ureteral invasion due to the progression of a cesarean scar pregnancy. *Case*. A 41-year-old woman, with a history of three cesarean sections and two miscarriages, was referred to our hospital at 25 weeks of gestation with a diagnosis of placenta accreta spectrum and bladder invasion. Although the gestational sac was located anterior to the lower uterine segment, a cesarean-scar pregnancy was not diagnosed. A cesarean hysterectomy was performed at 31 weeks of gestation with the placement of an aortic balloon. The placenta was found to adhere to the ureter with more than the expected parenchymal tissue displacement (FIGO Classification 3b). The ureter was not obstructed and was preserved by leaving the placenta slightly on the ureteral side. Postoperatively, a ureteral stent was placed because of the ureteral stricture in the area where the placenta had adhered. Two months after surgery, the ureteral stent was removed after observing an improvement in stenosis. An adherent placenta due to continued cesarean scar pregnancy should be managed by assuming placental invasion beyond the parenchyma into the ureter.

## 1. Introduction

Cesarean scar pregnancy (CSP) is the migration of blastocysts into the myometrium or into the defect of a scar from a prior cesarean birth. The mechanism of implantation in CSP is unknown, but it is thought that endogenous migration of the embryo through a wedge defect in the lower uterus or a microscopic fistula in the scar, invasion of placental villi into the uterine wall at the site of scar dissection, and hypoxia in the scar tissue induce implantation of the blastocyst. Although a cesarean scar defect occurs in approximately 1 in 2000 pregnancies, the incidence of CSP increases with the cesarean section rate [1–3]. The diagnosis of CSP is based on the presence of a gestational sac at the site of a previous uterine incision, an empty uterine cavity and cervix, and a thin myometrium adjacent to the bladder [4]. However, prenatal diagnosis of CSP is challenging, and it has been reported that 15.4% of CSPs were misdiagnosed as incomplete abortion or cervical pregnancy [2]. Termination is recommended because continued CSPs can result in adhesions to the placenta, hysterectomy, and heavy bleeding during delivery [4, 5]. Although the prevalence of bladder invasion in cases of continued CSP is not known, a previous study reported bladder injuries in 5 of the 10 cases of continued CSP and ureter injury in one of these five cases [6]. However, it was unclear whether the injury was caused by surgical complications or ureteral invasion of the placenta. Herein, we report a case of placenta accreta spectrum (PAS) in which the pregnancy continued without being diagnosed as CSP in the first trimester, resulting in the avoidance of ureteral resection despite the presence of bladder and ureteral invasions.

## 2. Case Presentation

**Figure 1:**
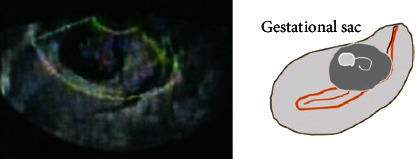
Ultrasound findings at 9 weeks' gestation at a previous clinic. The fetal sac was located in the lower part of the anterior wall of the uterus.

**Figure 2:**
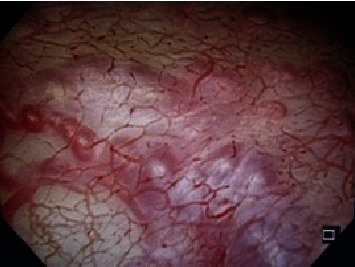
Cystoscopy findings at 26 weeks' gestation. Protrusions of blood vessels on the mucosa from near the bladder triangle to the posterior wall.

**Figure 3:**
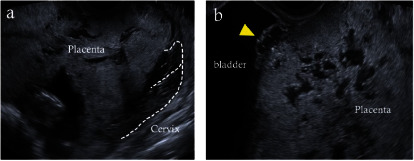
Ultrasound findings at 30 weeks of gestation. (a) Placental distension at the anterior uterine wall with a posterior deviation of the cervix. (b) Enlarged vascular protrusion (yellow triangle) and increased lacunae.

**Figure 4:**
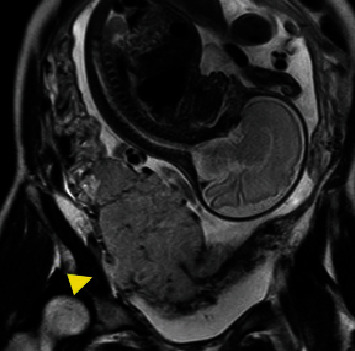
MRI T2 coronal section. The muscle layer on the right side of the lower uterine corpus appears to be replaced by the placenta (yellow triangle), but a ureteral invasion is not suspected. MRI, magnetic resonance imaging.

A 41-year-old woman, with a history of three cesarean sections and two miscarriages, visited her previous physician for a spontaneous pregnancy. Although a gestational sac was present in the lower uterus, it was not diagnosed as CSP (Figure 1). She was referred to our hospital at 25 weeks of gestation with suspected placenta praevia and was admitted to the hospital owing to suspected, threatened preterm delivery. At 26 weeks, cystoscopy revealed vascular protuberances in the mucosa from near the bladder triangle to the posterior wall (Figure 2). At 30 weeks, ultrasonography revealed a bulging placenta within the scar, posterior deviation of the cervical canal, and enlarged vessels protruding toward the bladder (Figure 3). Based on these findings, the patient was diagnosed with PAS invading the bladder due to the continuation of CSP. Magnetic resonance imaging showed vertical stretching of the bladder and placental protrusion towards the bladder, suggesting placental invasion into the bladder wall. The muscle layer on the right side of the lower uterine corpus appeared to have been replaced by the placenta. However, a ureteral invasion was not suspected (Figure 4). Additionally, there was no preoperative evidence of hydroureter formation. Repeat cesarean delivery between 34 0/7 and 35 6/7 weeks of gestation is recommended for patients who opt for expectant management of CSP [4]. However, in this case, the patient was considered at high risk for uterine rupture owing to the increased vascular protrusion into the bladder and persistent uterine contractions in the preceding weeks. We decided on an elective cesarean hysterectomy at 31 weeks and 6 days of gestation based on discussions with urologists, radiologists, anesthesiologists, and pediatric specialists. The newborn (female; birth weight, 1504 g; Apgar score 2/3/6) was delivered through a transverse incision in the uterine fundus under general anesthesia after the placement of a ureteral stent. Subsequently, bilateral uterine and bladder artery embolization was performed. Cystectomy was deemed unnecessary because the bladder artery had been embolized beforehand and bleeding from the site of placental perforation was thought to be controllable. The bladder was dissected by tying off the cervix using a *nelaton catheter*, leaving the placenta slightly on the bladder. However, several arterial hemorrhages were observed in the remaining villi in the bladder, and hemostasis was difficult. The placenta adhered to the ureter and replaced the parenchyma. The uterus was then removed, leaving a few villi attached to the ureter. Bleeding from villi adherent to the ureter may be effectively controlled using a bipolar coagulator.

**Figure 5:**
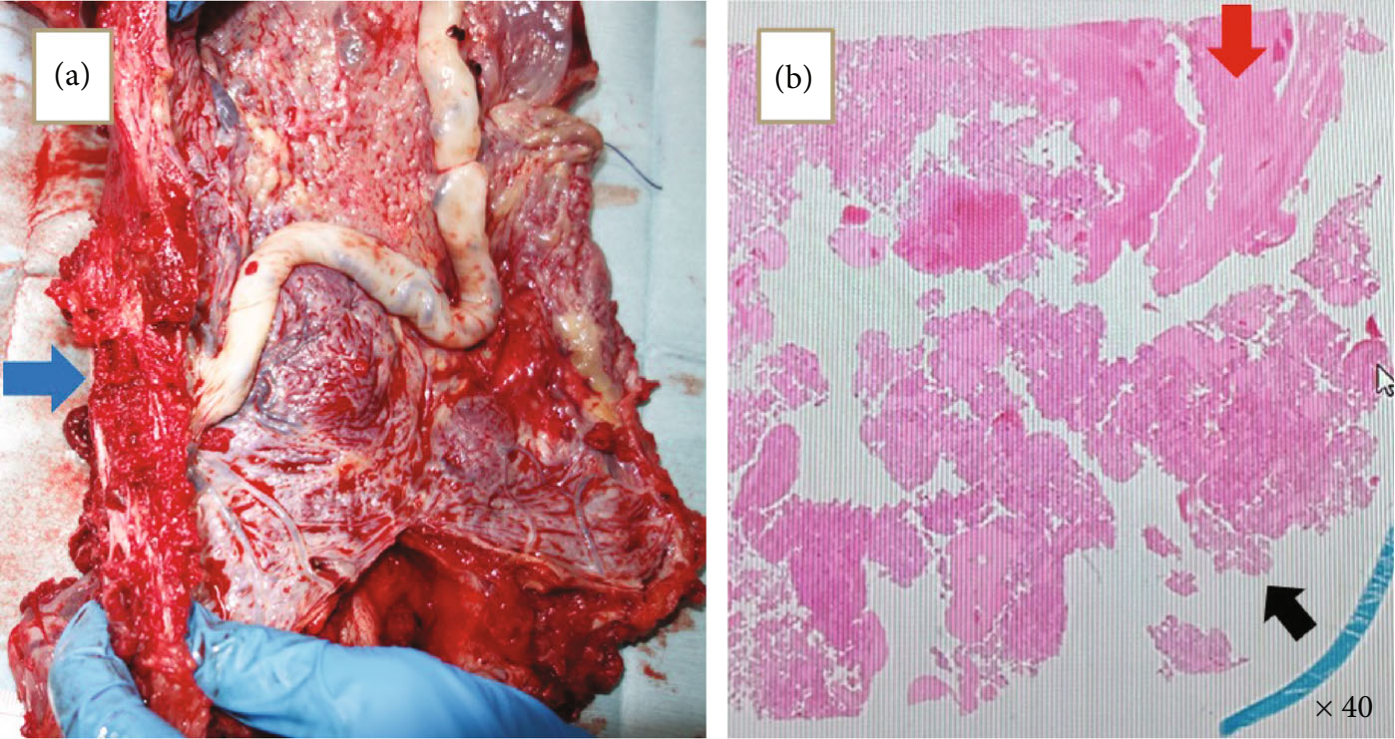
Gross and pathological findings of the placenta. (a) Gross findings of the placenta show that the placenta extends to the serosa (blue arrow). (b) Placental pathology showed chorionic components on the serous surface (red arrow) through the myometrium (black arrow).

An aortic balloon was placed but was not inflated. Intraoperative blood loss was 6600 mL, and 900 mL of autologous RBC (red blood cells), 967 mL of autologous fresh-frozen plasma (FFP), 8 units of RBC, 12 units of FFP, 12 units of cryoprecipitate, and 20 units of platelets were transfused. Gross findings of the placenta show that the placenta extends to the serosa. Placental pathology showed chorionic components on the serous surface through the myometrium. (Figure 5).

The ureteral stent was removed on postoperative day 22, although resection was performed due to stenosis in the right ureter. On postoperative day 64, the stenosis had improved, and the right ureteral stent was removed.

## 3. Discussion

The birth rates among patients with CSP with expectant management and hysterectomy with placenta accreta are 57% and 63%, respectively [5]. Therefore, termination is often recommended for CSP due to the significant risk of maternal morbidity. [4]. The implantation pattern of CSP can be divided into endogenic (also called “on the scar”) and exogenic (also called “in the niche”) [7]. Endogenic CSP can be particularly difficult to differentiate from the low implantation of an intrauterine pregnancy. In a literature review of 751 CSP cases, 107 (13.6%) were misdiagnosed as cervical ectopic pregnancy, spontaneous abortion, or low implantation of an intrauterine pregnancy [8]. In the present case, the placenta was located in the lower part of the uterus in the early stages of pregnancy but was followed up without being diagnosed with CSP. It is important to differentiate CSP in early pregnancy and assess the risk of PAS and uterine rupture.

One case of ureteral injury was reported in 10 patients with PAS who underwent expectant management of CSP [6]. Furthermore, the urinary tract injury frequency in morbidly adherent placentas is reported to be 21.7%, of which bladder injuries account for 11.7%, ureteral injuries for 4.7%, and bladder with ureteral injuries for 5.3% [9]. In a previously reported study, 11.1% of adherent placentas involved the ureter and required ureteric reimplantation [10], and the ureteral stent was not placed preoperatively in a patient, resulting in ureteral injury requiring postoperative ureteric reimplantation [10]. However, the use of ureteral stents in association with total cesarean hysterectomy was reported not to reduce the risk of ureteral injury [11]. A national survey in the U.S. showed high maternal morbidity and mortality from placenta percreta, with a severe maternal morbidity of 82.1% and mortality of 1.4% [12]. In these patients, urinary cystectomy was performed in 6.1% [12]. These risks may be mitigated by early referral to a PAS center of excellence.

A case report describes an intraoperative identification of a hydroureter due to ureteral invasion of the placenta, resulting in ureteroneocystostomy [13]. In our patient, there was no preoperative evidence of hydroureter formation. Although the postoperative placement of a stent was required for ureteral stenosis, spontaneous resolution of the stenosis was achieved, suggesting that the stenosis of the ureter may have been mild. This suggests that ureteral preservation can be determined by the presence or absence of a hydroureter. Therefore, it is important to suspect preoperative invasion of the ureter and confirm the existence of a hydroureter.

It is unclear whether ureteral injuries were due to placental invasion or surgical complications. In a recent systematic review, the placenta was presumed to not invade the ureter or exceed the limits of the uterine serosa [14, 15]. Rather, the placenta and ureter may appear to be in close proximity and adhesion due to anatomic distortion caused by a bulging placenta, thinning of the myometrium, and past adhesions from surgery. Since these reports still cover only a small number of cases, with even fewer cases of grade 3, further analysis of a large number of cases is required. Moreover, even if this is the case pathologically, it should be assumed as an adhesion or invasion into the ureter clinically and managed individually.

Milenda et al. reported that uterine artery embolization prior to a hysterectomy after cesarean section is safe and effective in reducing the blood loss, need for blood transfusion, and length of ICU stay, compared with cesarean hysterectomy alone [16]. Despite the preemptive embolization of the bladder artery prior to bladder dissection, we observed persistent and significant arterial bleeding from the surface of the bladder dissection. The rapid blood flow in the bladder artery may have rendered embolization insufficient in this case, leading to continued bleeding. However, cervical tourniquets were useful in decreasing the amount of bleeding.

To conclude, an adherent placenta due to continued CSP should be managed by assuming placental invasion beyond the parenchyma into the ureter. Preoperative identification of the hydroureter is essential to confirm ureteral invasion in CSP. To prevent massive intraoperative hemorrhage and organ damage, it is necessary to collaborate with multiple departments including obstetrics and gynecology, anesthesiology, radiology, urology, and pediatrics.

## Data Availability

Data supporting this research article are available from the corresponding author on reasonable request.
